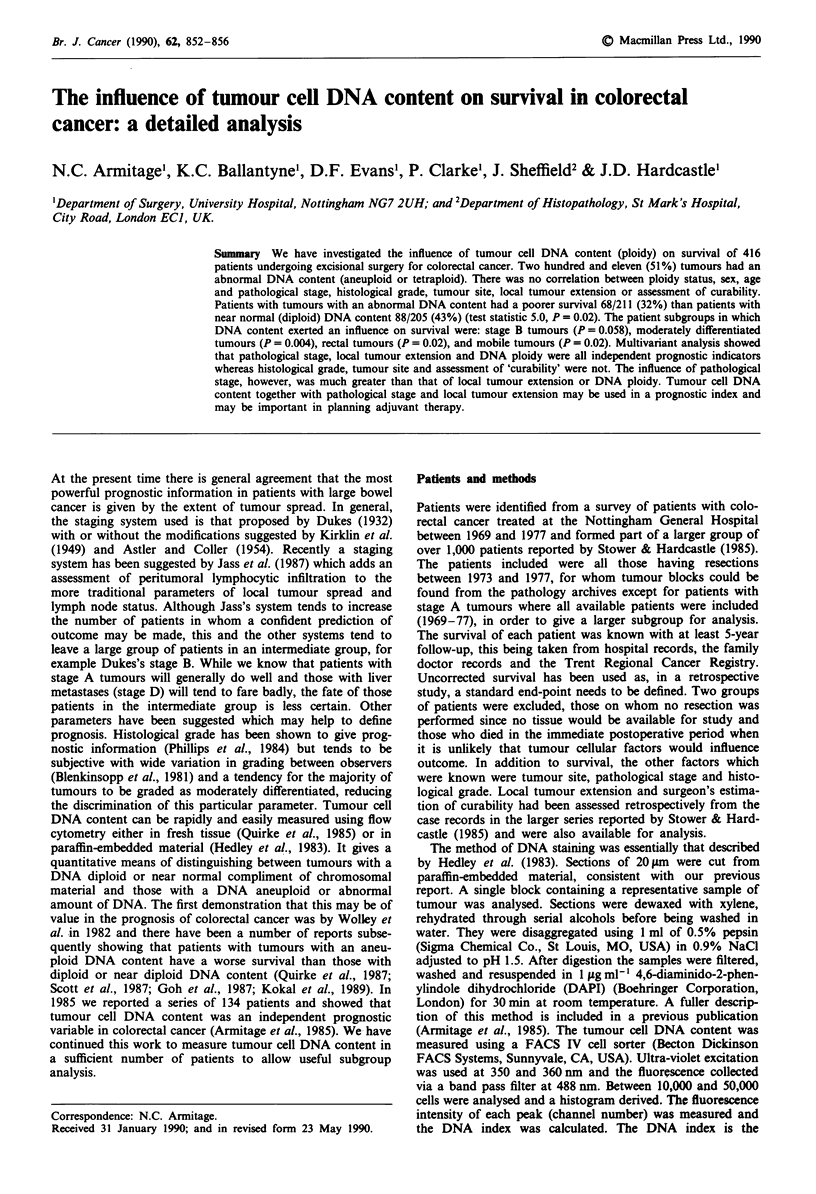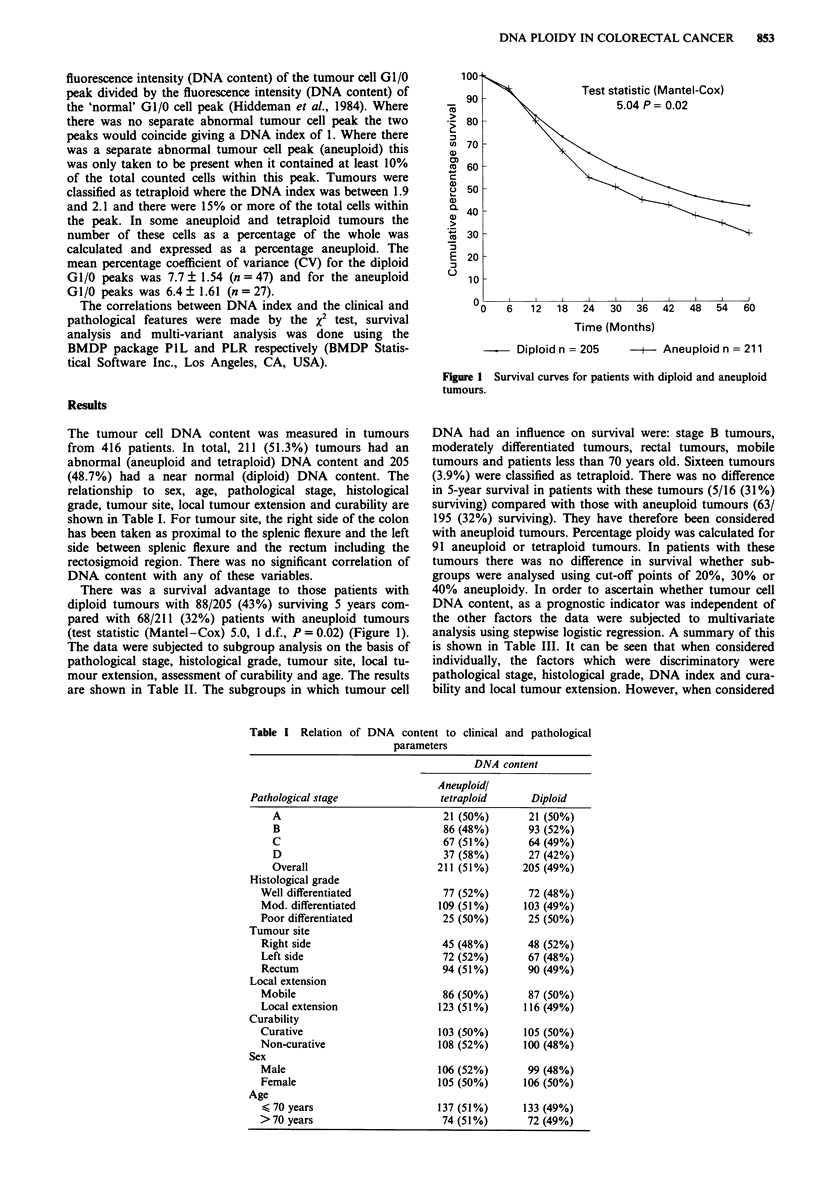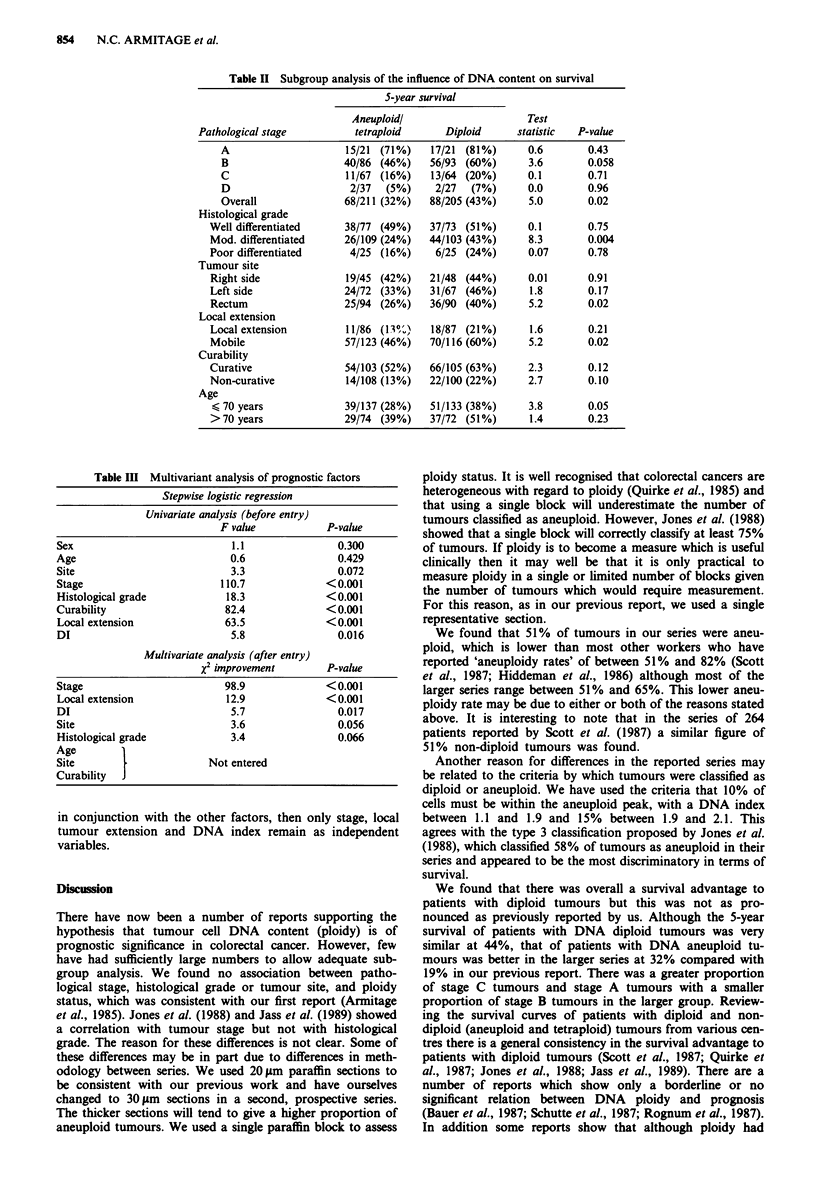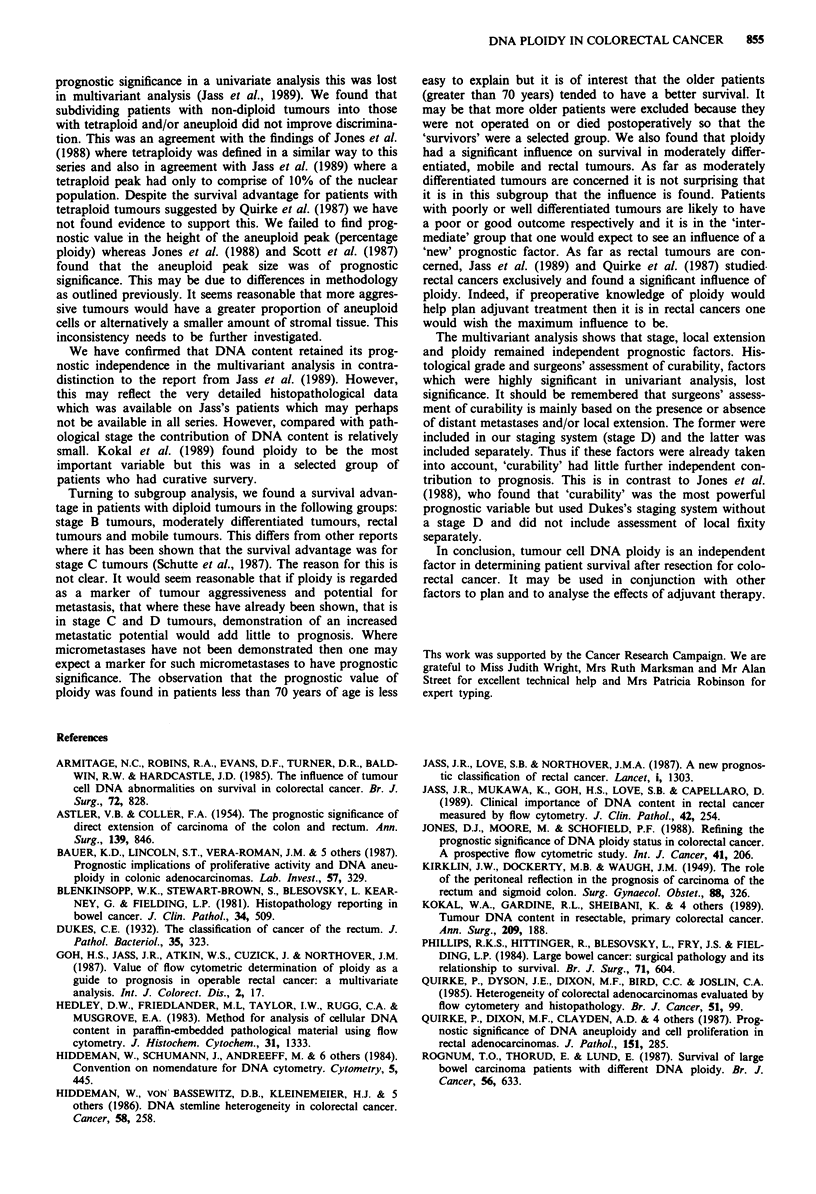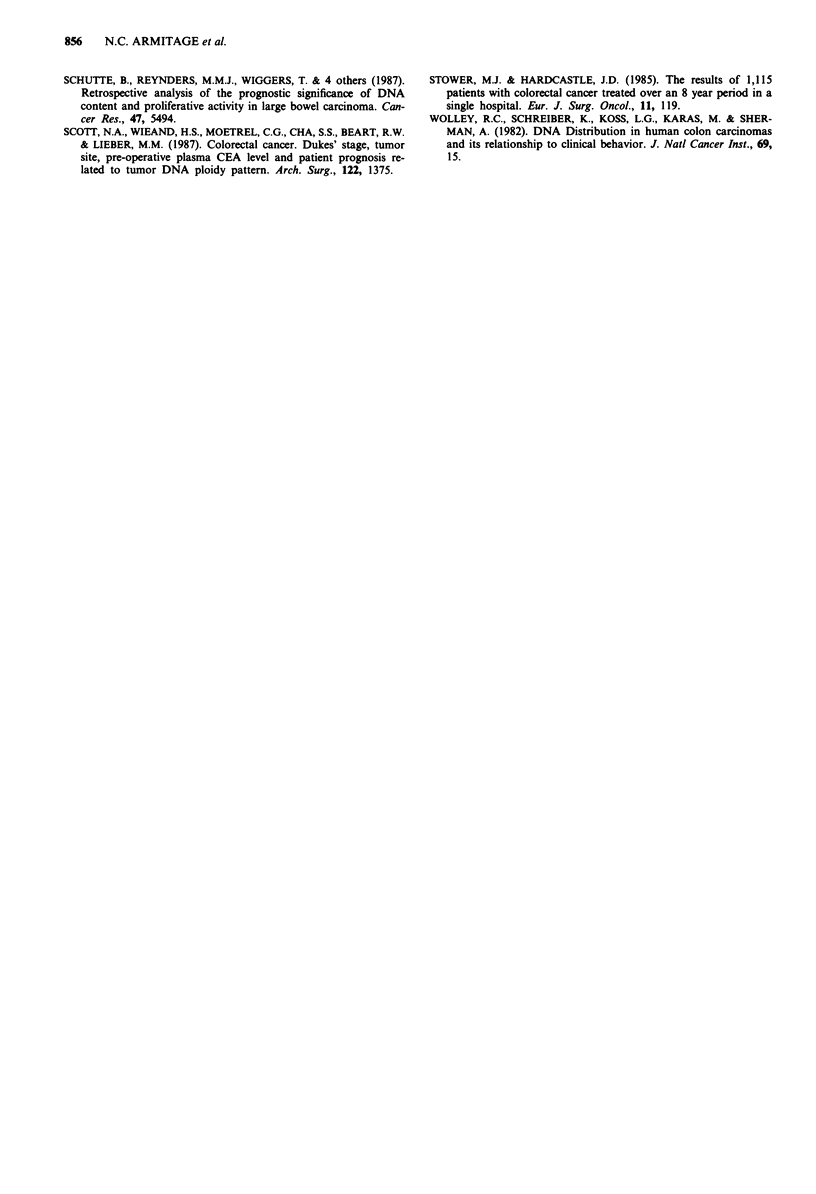# The influence of tumour cell DNA content on survival in colorectal cancer: a detailed analysis.

**DOI:** 10.1038/bjc.1990.393

**Published:** 1990-11

**Authors:** N. C. Armitage, K. C. Ballantyne, D. F. Evans, P. Clarke, J. Sheffield, J. D. Hardcastle

**Affiliations:** Department of Surgery, University Hospital, Nottingham, UK.

## Abstract

We have investigated the influence of tumour cell DNA content (ploidy) on survival of 416 patients undergoing excisional surgery for colorectal cancer. Two hundred and eleven (51%) tumours had an abnormal DNA content (aneuploid or tetraploid). There was no correlation between ploidy status, sex, age and pathological stage, histological grade, tumour site, local tumour extension or assessment of curability. Patients with tumours with an abnormal DNA content had a poorer survival 68/211 (32%) than patients with near normal (diploid) DNA content 88/205 (43%) (test statistic 5.0, P = 0.02). The patient subgroups in which DNA content exerted an influence on survival were: stage B tumours (P = 0.0058), moderately differentiated tumours (P = 0.004), rectal tumours (P = 0.02), and mobile tumours (P = 0.02). Multivariant analysis showed that pathological stage, local tumour extension and DNA ploidy were all independent prognostic indicators whereas histological grade, tumour site and assessment of 'curability' were not. The influence of pathological stage, however, was much greater than that of local tumor extension or DNA ploidy. Tumour cell DNA content together with pathological stage and local tumour extension may be used in a prognostic index and may be important in planning adjuvant therapy.


Br. J. Cancer (1990), 62, 852-856 © Macmillan Press Ltd., 1990

### The influence of tumour cell DNA content on survival in colorectal cancer: a detailed analysis

N.C. Armitage[1], K.C. Ballantyne[1], D.F. Evans[1], P. Clarke[1], J. Sheffield[2] & J.D. Hardcastle[1]

[1]*Department of Surgery, University Hospital, Nottingham NG7 2UH; and* [2]*Department of Histopathology, St Mark's Hospital, City Road, London EC1, UK.*

**Summary** We have investigated the influence of tumour cell DNA content (ploidy) on survival of 416 patients undergoing excisional surgery for colorectal cancer. Two hundred and eleven (51%) tumours had an abnormal DNA content (aneuploid or tetraploid). There was no correlation between ploidy status, sex, age and pathological stage, histological grade, tumour site, local tumour extension or assessment of curability. Patients with tumours with an abnormal DNA content had a poorer survival 68/211 (32%) than patients with near normal (diploid) DNA content 88/205 (43%) (test statistic 5.0, $P = 0.02$). The patient subgroups in which DNA content exerted an influence on survival were: stage B tumours ($P = 0.058$), moderately differentiated tumours ($P = 0.004$), rectal tumours ($P = 0.02$), and mobile tumours ($P = 0.02$). Multivariant analysis showed that pathological stage, local tumour extension and DNA ploidy were all independent prognostic indicators whereas histological grade, tumour site and assessment of 'curability' were not. The influence of pathological stage, however, was much greater than that of local tumour extension or DNA ploidy. Tumour cell DNA content together with pathological stage and local tumour extension may be used in a prognostic index and may be important in planning adjuvant therapy.

At the present time there is general agreement that the most powerful prognostic information in patients with large bowel cancer is given by the extent of tumour spread. In general, the staging system used is that proposed by Dukes (1932) with or without the modifications suggested by Kirklin *et al.* (1949) and Astler and Coller (1954). Recently a staging system has been suggested by Jass *et al.* (1987) which adds an assessment of peritumoral lymphocytic infiltration to the more traditional parameters of local tumour spread and lymph node status. Although Jass's system tends to increase the number of patients in whom a confident prediction of outcome may be made, this and the other systems tend to leave a large group of patients in an intermediate group, for example Dukes's stage B. While we know that patients with stage A tumours will generally do well and those with liver metastases (stage D) will tend to fare badly, the fate of those patients in the intermediate group is less certain. Other parameters have been suggested which may help to define prognosis. Histological grade has been shown to give prognostic information (Phillips *et al.*, 1984) but tends to be subjective with wide variation in grading between observers (Blenkinsopp *et al.*, 1981) and a tendency for the majority of tumours to be graded as moderately differentiated, reducing the discrimination of this particular parameter. Tumour cell DNA content can be rapidly and easily measured using flow cytometry either in fresh tissue (Quirke *et al.*, 1985) or in paraffin-embedded material (Hedley *et al.*, 1983). It gives a quantitative means of distinguishing between tumours with a DNA diploid or near normal compliment of chromosomal material and those with a DNA aneuploid or abnormal amount of DNA. The first demonstration that this may be of value in the prognosis of colorectal cancer was by Wolley *et al.* in 1982 and there have been a number of reports subsequently showing that patients with tumours with an aneuploid DNA content have a worse survival than those with diploid or near diploid DNA content (Quirke *et al.*, 1987; Scott *et al.*, 1987; Goh *et al.*, 1987; Kokal *et al.*, 1989). In 1985 we reported a series of 134 patients and showed that tumour cell DNA content was an independent prognostic variable in colorectal cancer (Armitage *et al.*, 1985). We have continued this work to measure tumour cell DNA content in a sufficient number of patients to allow useful subgroup analysis.

Correspondence: N.C. Armitage.
Received 31 January 1990; and in revised form 23 May 1990.

### Patients and methods

Patients were identified from a survey of patients with colorectal cancer treated at the Nottingham General Hospital between 1969 and 1977 and formed part of a larger group of over 1,000 patients reported by Stower & Hardcastle (1985). The patients included were all those having resections between 1973 and 1977, for whom tumour blocks could be found from the pathology archives except for patients with stage A tumours where all available patients were included (1969–77), in order to give a larger subgroup for analysis. The survival of each patient was known with at least 5-year follow-up, this being taken from hospital records, the family doctor records and the Trent Regional Cancer Registry. Uncorrected survival has been used as, in a retrospective study, a standard end-point needs to be defined. Two groups of patients were excluded, those on whom no resection was performed since no tissue would be available for study and those who died in the immediate postoperative period when it is unlikely that tumour cellular factors would influence outcome. In addition to survival, the other factors which were known were tumour site, pathological stage and histological grade. Local tumour extension and surgeon's estimation of curability had been assessed retrospectively from the case records in the larger series reported by Stower & Hardcastle (1985) and were also available for analysis.

The method of DNA staining was essentially that described by Hedley *et al.* (1983). Sections of 20 μm were cut from paraffin-embedded material, consistent with our previous report. A single block containing a representative sample of tumour was analysed. Sections were dewaxed with xylene, rehydrated through serial alcohols before being washed in water. They were disaggregated using 1 ml of 0.5% pepsin (Sigma Chemical Co., St Louis, MO, USA) in 0.9% NaCl adjusted to pH 1.5. After digestion the samples were filtered, washed and resuspended in 1 $\mu g\,ml^{-1}$ 4,6-diaminido-2-phenylindole dihydrochloride (DAPI) (Boehringer Corporation, London) for 30 min at room temperature. A fuller description of this method is included in a previous publication (Armitage *et al.*, 1985). The tumour cell DNA content was measured using a FACS IV cell sorter (Becton Dickinson FACS Systems, Sunnyvale, CA, USA). Ultra-violet excitation was used at 350 and 360 nm and the fluorescence collected via a band pass filter at 488 nm. Between 10,000 and 50,000 cells were analysed and a histogram derived. The fluorescence intensity of each peak (channel number) was measured and the DNA index was calculated. The DNA index is the fluorescence intensity (DNA content) of the tumour cell G1/0 peak divided by the fluorescence intensity (DNA content) of the 'normal' G1/0 cell peak (Hiddeman *et al.*, 1984). Where there was no separate abnormal tumour cell peak the two peaks would coincide giving a DNA index of 1. Where there was a separate abnormal tumour cell peak (aneuploid) this was only taken to be present when it contained at least 10% of the total counted cells within this peak. Tumours were classified as tetraploid where the DNA index was between 1.9 and 2.1 and there were 15% or more of the total cells within the peak. In some aneuploid and tetraploid tumours the number of these cells as a percentage of the whole was calculated and expressed as a percentage aneuploid. The mean percentage coefficient of variance (CV) for the diploid G1/0 peaks was 7.7 ± 1.54 ($n = 47$) and for the aneuploid G1/0 peaks was 6.4 ± 1.61 ($n = 27$).

The correlations between DNA index and the clinical and pathological features were made by the $\chi^2$ test, survival analysis and multi-variant analysis was done using the BMDP package P1L and PLR respectively (BMDP Statistical Software Inc., Los Angeles, CA, USA).

### Results

The tumour cell DNA content was measured in tumours from 416 patients. In total, 211 (51.3%) tumours had an abnormal (aneuploid and tetraploid) DNA content and 205 (48.7%) had a near normal (diploid) DNA content. The relationship to sex, age, pathological stage, histological grade, tumour site, local tumour extension and curability are shown in Table I. For tumour site, the right side of the colon has been taken as proximal to the splenic flexure and the left side between splenic flexure and the rectum including the rectosigmoid region. There was no significant correlation of DNA content with any of these variables.

There was a survival advantage to those patients with diploid tumours with 88/205 (43%) surviving 5 years compared with 68/211 (32%) patients with aneuploid tumours (test statistic (Mantel-Cox) 5.0, 1 d.f., $P = 0.02$) (Figure 1). The data were subjected to subgroup analysis on the basis of pathological stage, histological grade, tumour site, local tumour extension, assessment of curability and age. The results are shown in Table II. The subgroups in which tumour cell DNA had an influence on survival were: stage B tumours, moderately differentiated tumours, rectal tumours, mobile tumours and patients less than 70 years old. Sixteen tumours (3.9%) were classified as tetraploid. There was no difference in 5-year survival in patients with these tumours (5/16 (31%) surviving) compared with those with aneuploid tumours (63/195 (32%) surviving). They have therefore been considered with aneuploid tumours. Percentage ploidy was calculated for 91 aneuploid or tetraploid tumours. In patients with these tumours there was no difference in survival whether subgroups were analysed using cut-off points of 20%, 30% or 40% aneuploidy. In order to ascertain whether tumour cell DNA content, as a prognostic indicator was independent of the other factors the data were subjected to multivariate analysis using stepwise logistic regression. A summary of this is shown in Table III. It can be seen that when considered individually, the factors which were discriminatory were pathological stage, histological grade, DNA index and curability and local tumour extension. However, when considered

**Figure 1** Survival curves for patients with diploid and aneuploid tumours.

**Table I** Relation of DNA content to clinical and pathological parameters

| | *DNA content* | |
|---|---|---|
| *Pathological stage* | *Aneuploid/tetraploid* | *Diploid* |
| A | 21 (50%) | 21 (50%) |
| B | 86 (48%) | 93 (52%) |
| C | 67 (51%) | 64 (49%) |
| D | 37 (58%) | 27 (42%) |
| Overall | 211 (51%) | 205 (49%) |
| Histological grade | | |
| Well differentiated | 77 (52%) | 72 (48%) |
| Mod. differentiated | 109 (51%) | 103 (49%) |
| Poor differentiated | 25 (50%) | 25 (50%) |
| Tumour site | | |
| Right side | 45 (48%) | 48 (52%) |
| Left side | 72 (52%) | 67 (48%) |
| Rectum | 94 (51%) | 90 (49%) |
| Local extension | | |
| Mobile | 86 (50%) | 87 (50%) |
| Local extension | 123 (51%) | 116 (49%) |
| Curability | | |
| Curative | 103 (50%) | 105 (50%) |
| Non-curative | 108 (52%) | 100 (48%) |
| Sex | | |
| Male | 106 (52%) | 99 (48%) |
| Female | 105 (50%) | 106 (50%) |
| Age | | |
| ≤ 70 years | 137 (51%) | 133 (49%) |
| > 70 years | 74 (51%) | 72 (49%) |

**Table II** Subgroup analysis of the influence of DNA content on survival

| | *5-year survival* | | | |
|---|---|---|---|---|
| *Pathological stage* | *Aneuploid/ tetraploid* | *Diploid* | *Test statistic* | *P-value* |
| A | 15/21 (71%) | 17/21 (81%) | 0.6 | 0.43 |
| B | 40/86 (46%) | 56/93 (60%) | 3.6 | 0.058 |
| C | 11/67 (16%) | 13/64 (20%) | 0.1 | 0.71 |
| D | 2/37 (5%) | 2/27 (7%) | 0.0 | 0.96 |
| Overall | 68/211 (32%) | 88/205 (43%) | 5.0 | 0.02 |
| Histological grade | | | | |
| Well differentiated | 38/77 (49%) | 37/73 (51%) | 0.1 | 0.75 |
| Mod. differentiated | 26/109 (24%) | 44/103 (43%) | 8.3 | 0.004 |
| Poor differentiated | 4/25 (16%) | 6/25 (24%) | 0.07 | 0.78 |
| Tumour site | | | | |
| Right side | 19/45 (42%) | 21/48 (44%) | 0.01 | 0.91 |
| Left side | 24/72 (33%) | 31/67 (46%) | 1.8 | 0.17 |
| Rectum | 25/94 (26%) | 36/90 (40%) | 5.2 | 0.02 |
| Local extension | | | | |
| Local extension | 11/86 (13%) | 18/87 (21%) | 1.6 | 0.21 |
| Mobile | 57/123 (46%) | 70/116 (60%) | 5.2 | 0.02 |
| Curability | | | | |
| Curative | 54/103 (52%) | 66/105 (63%) | 2.3 | 0.12 |
| Non-curative | 14/108 (13%) | 22/100 (22%) | 2.7 | 0.10 |
| Age | | | | |
| $\leqslant$ 70 years | 39/137 (28%) | 51/133 (38%) | 3.8 | 0.05 |
| > 70 years | 29/74 (39%) | 37/72 (51%) | 1.4 | 0.23 |

**Table III** Multivariant analysis of prognostic factors

| | *Stepwise logistic regression* | |
|---|---|---|
| | *Univariate analysis (before entry)* *F value* | *P-value* |
| Sex | 1.1 | 0.300 |
| Age | 0.6 | 0.429 |
| Site | 3.3 | 0.072 |
| Stage | 110.7 | <0.001 |
| Histological grade | 18.3 | <0.001 |
| Curability | 82.4 | <0.001 |
| Local extension | 63.5 | <0.001 |
| DI | 5.8 | 0.016 |
| | *Multivariate analysis (after entry)* $\chi^2$ *improvement* | *P-value* |
| Stage | 98.9 | <0.001 |
| Local extension | 12.9 | <0.001 |
| DI | 5.7 | 0.017 |
| Site | 3.6 | 0.056 |
| Histological grade | 3.4 | 0.066 |
| Age, Site, Curability | Not entered | |

in conjunction with the other factors, then only stage, local tumour extension and DNA index remain as independent variables.

### Discussion

There have now been a number of reports supporting the hypothesis that tumour cell DNA content (ploidy) is of prognostic significance in colorectal cancer. However, few have had sufficiently large numbers to allow adequate subgroup analysis. We found no association between pathological stage, histological grade or tumour site, and ploidy status, which was consistent with our first report (Armitage *et al.*, 1985). Jones *et al.* (1988) and Jass *et al.* (1989) showed a correlation with tumour stage but not with histological grade. The reason for these differences is not clear. Some of these differences may be in part due to differences in methodology between series. We used 20 µm paraffin sections to be consistent with our previous work and have ourselves changed to 30 µm sections in a second, prospective series. The thicker sections will tend to give a higher proportion of aneuploid tumours. We used a single paraffin block to assess ploidy status. It is well recognised that colorectal cancers are heterogeneous with regard to ploidy (Quirke *et al.*, 1985) and that using a single block will underestimate the number of tumours classified as aneuploid. However, Jones *et al.* (1988) showed that a single block will correctly classify at least 75% of tumours. If ploidy is to become a measure which is useful clinically then it may well be that it is only practical to measure ploidy in a single or limited number of blocks given the number of tumours which would require measurement. For this reason, as in our previous report, we used a single representative section.

We found that 51% of tumours in our series were aneuploid, which is lower than most other workers who have reported 'aneuploidy rates' of between 51% and 82% (Scott *et al.*, 1987; Hiddeman *et al.*, 1986) although most of the larger series range between 51% and 65%. This lower aneuploidy rate may be due to either or both of the reasons stated above. It is interesting to note that in the series of 264 patients reported by Scott *et al.* (1987) a similar figure of 51% non-diploid tumours was found.

Another reason for differences in the reported series may be related to the criteria by which tumours were classified as diploid or aneuploid. We have used the criteria that 10% of cells must be within the aneuploid peak, with a DNA index between 1.1 and 1.9 and 15% between 1.9 and 2.1. This agrees with the type 3 classification proposed by Jones *et al.* (1988), which classified 58% of tumours as aneuploid in their series and appeared to be the most discriminatory in terms of survival.

We found that there was overall a survival advantage to patients with diploid tumours but this was not as pronounced as previously reported by us. Although the 5-year survival of patients with DNA diploid tumours was very similar at 44%, that of patients with DNA aneuploid tumours was better in the larger series at 32% compared with 19% in our previous report. There was a greater proportion of stage C tumours and stage A tumours with a smaller proportion of stage B tumours in the larger group. Reviewing the survival curves of patients with diploid and non-diploid (aneuploid and tetraploid) tumours from various centres there is a general consistency in the survival advantage to patients with diploid tumours (Scott *et al.*, 1987; Quirke *et al.*, 1987; Jones *et al.*, 1988; Jass *et al.*, 1989). There are a number of reports which show only a borderline or no significant relation between DNA ploidy and prognosis (Bauer *et al.*, 1987; Schutte *et al.*, 1987; Rognum *et al.*, 1987). In addition some reports show that although ploidy had prognostic significance in a univariate analysis this was lost in multivariant analysis (Jass *et al.*, 1989). We found that subdividing patients with non-diploid tumours into those with tetraploid and/or aneuploid did not improve discrimination. This was an agreement with the findings of Jones *et al.* (1988) where tetraploidy was defined in a similar way to this series and also in agreement with Jass *et al.* (1989) where a tetraploid peak had only to comprise of 10% of the nuclear population. Despite the survival advantage for patients with tetraploid tumours suggested by Quirke *et al.* (1987) we have not found evidence to support this. We failed to find prognostic value in the height of the aneuploid peak (percentage ploidy) whereas Jones *et al.* (1988) and Scott *et al.* (1987) found that the aneuploid peak size was of prognostic significance. This may be due to differences in methodology as outlined previously. It seems reasonable that more aggressive tumours would have a greater proportion of aneuploid cells or alternatively a smaller amount of stromal tissue. This inconsistency needs to be further investigated.

We have confirmed that DNA content retained its prognostic independence in the multivariant analysis in contradistinction to the report from Jass *et al.* (1989). However, this may reflect the very detailed histopathological data which was available on Jass's patients which may perhaps not be available in all series. However, compared with pathological stage the contribution of DNA content is relatively small. Kokal *et al.* (1989) found ploidy to be the most important variable but this was in a selected group of patients who had curative survery.

Turning to subgroup analysis, we found a survival advantage in patients with diploid tumours in the following groups: stage B tumours, moderately differentiated tumours, rectal tumours and mobile tumours. This differs from other reports where it has been shown that the survival advantage was for stage C tumours (Schutte *et al.*, 1987). The reason for this is not clear. It would seem reasonable that if ploidy is regarded as a marker of tumour aggressiveness and potential for metastasis, that where these have already been shown, that is in stage C and D tumours, demonstration of an increased metastatic potential would add little to prognosis. Where micrometastases have not been demonstrated then one may expect a marker for such micrometastases to have prognostic significance. The observation that the prognostic value of ploidy was found in patients less than 70 years of age is less easy to explain but it is of interest that the older patients (greater than 70 years) tended to have a better survival. It may be that more older patients were excluded because they were not operated on or died postoperatively so that the 'survivors' were a selected group. We also found that ploidy had a significant influence on survival in moderately differentiated, mobile and rectal tumours. As far as moderately differentiated tumours are concerned it is not surprising that it is in this subgroup that the influence is found. Patients with poorly or well differentiated tumours are likely to have a poor or good outcome respectively and it is in the 'intermediate' group that one would expect to see an influence of a 'new' prognostic factor. As far as rectal tumours are concerned, Jass *et al.* (1989) and Quirke *et al.* (1987) studied rectal cancers exclusively and found a significant influence of ploidy. Indeed, if preoperative knowledge of ploidy would help plan adjuvant treatment then it is in rectal cancers one would wish the maximum influence to be.

The multivariant analysis shows that stage, local extension and ploidy remained independent prognostic factors. Histological grade and surgeons' assessment of curability, factors which were highly significant in univariant analysis, lost significance. It should be remembered that surgeons' assessment of curability is mainly based on the presence or absence of distant metastases and/or local extension. The former were included in our staging system (stage D) and the latter was included separately. Thus if these factors were already taken into account, 'curability' had little further independent contribution to prognosis. This is in contrast to Jones *et al.* (1988), who found that 'curability' was the most powerful prognostic variable but used Dukes's staging system without a stage D and did not include assessment of local fixity separately.

In conclusion, tumour cell DNA ploidy is an independent factor in determining patient survival after resection for colorectal cancer. It may be used in conjunction with other factors to plan and to analyse the effects of adjuvant therapy.

Ths work was supported by the Cancer Research Campaign. We are grateful to Miss Judith Wright, Mrs Ruth Marksman and Mr Alan Street for excellent technical help and Mrs Patricia Robinson for expert typing.

### References

ARMITAGE, N.C., ROBINS, R.A., EVANS, D.F., TURNER, D.R., BALDWIN, R.W. & HARDCASTLE, J.D. (1985). The influence of tumour cell DNA abnormalities on survival in colorectal cancer. *Br. J. Surg.*, **72**, 828.

ASTLER, V.B. & COLLER, F.A. (1954). The prognostic significance of direct extension of carcinoma of the colon and rectum. *Ann. Surg.*, **139**, 846.

BAUER, K.D., LINCOLN, S.T., VERA-ROMAN, J.M. & 5 others (1987). Prognostic implications of proliferative activity and DNA aneuploidy in colonic adenocarcinomas. *Lab. Invest.*, **57**, 329.

BLENKINSOPP, W.K., STEWART-BROWN, S., BLESOVSKY, L. KEARNEY, G. & FIELDING, L.P. (1981). Histopathology reporting in bowel cancer. *J. Clin. Pathol.*, **34**, 509.

DUKES, C.E. (1932). The classification of cancer of the rectum. *J. Pathol. Bacteriol.*, **35**, 323.

GOH, H.S., JASS, J.R., ATKIN, W.S., CUZICK, J. & NORTHOVER, J.M. (1987). Value of flow cytometric determination of ploidy as a guide to prognosis in operable rectal cancer: a multivariate analysis. *Int. J. Colorect. Dis.*, **2**, 17.

HEDLEY, D.W., FRIEDLANDER, M.L, TAYLOR, I.W., RUGG, C.A. & MUSGROVE, E.A. (1983). Method for analysis of cellular DNA content in paraffin-embedded pathological material using flow cytometry. *J. Histochem. Cytochem.*, **31**, 1333.

HIDDEMAN, W., SCHUMANN, J., ANDREEFF, M. & 6 others (1984). Convention on nomendature for DNA cytometry. *Cytometry*, **5**, 445.

HIDDEMAN, W., VON BASSEWITZ, D.B., KLEINEMEIER, H.J. & 5 others (1986). DNA stemline heterogeneity in colorectal cancer. *Cancer*, **58**, 258.

JASS, J.R., LOVE, S.B. & NORTHOVER, J.M.A. (1987). A new prognostic classification of rectal cancer. *Lancet*, **i**, 1303.

JASS, J.R., MUKAWA, K., GOH, H.S., LOVE, S.B. & CAPELLARO, D. (1989). Clinical importance of DNA content in rectal cancer measured by flow cytometry. *J. Clin. Pathol.*, **42**, 254.

JONES, D.J., MOORE, M. & SCHOFIELD, P.F. (1988). Refining the prognostic significance of DNA ploidy status in colorectal cancer. A prospective flow cytometric study. *Int. J. Cancer*, **41**, 206.

KIRKLIN, J.W., DOCKERTY, M.B. & WAUGH, J.M. (1949). The role of the peritoneal reflection in the prognosis of carcinoma of the rectum and sigmoid colon. *Surg. Gynaecol. Obstet.*, **88**, 326.

KOKAL, W.A., GARDINE, R.L., SHEIBANI, K. & 4 others (1989). Tumour DNA content in resectable, primary colorectal cancer. *Ann. Surg.*, **209**, 188.

PHILLIPS, R.K.S., HITTINGER, R., BLESOVSKY, L., FRY, J.S. & FIELDING, L.P. (1984). Large bowel cancer: surgical pathology and its relationship to survival. *Br. J. Surg.*, **71**, 604.

QUIRKE, P., DYSON, J.E., DIXON, M.F., BIRD, C.C. & JOSLIN, C.A. (1985). Heterogeneity of colorectal adenocarcinomas evaluated by flow cytometery and histopathology. *Br. J. Cancer*, **51**, 99.

QUIRKE, P., DIXON, M.F., CLAYDEN, A.D. & 4 others (1987). Prognostic significance of DNA aneuploidy and cell proliferation in rectal adenocarcinomas. *J. Pathol.*, **151**, 285.

ROGNUM, T.O., THORUD, E. & LUND, E. (1987). Survival of large bowel carcinoma patients with different DNA ploidy. *Br. J. Cancer*, **56**, 633.

SCHUTTE, B., REYNDERS, M.M.J., WIGGERS, T. & 4 others (1987). Retrospective analysis of the prognostic significance of DNA content and proliferative activity in large bowel carcinoma. *Cancer Res.*, **47**, 5494.

SCOTT, N.A., WIEAND, H.S., MOETREL, C.G., CHA, S.S., BEART, R.W. & LIEBER, M.M. (1987). Colorectal cancer. Dukes' stage, tumor site, pre-operative plasma CEA level and patient prognosis related to tumor DNA ploidy pattern. *Arch. Surg.*, **122**, 1375.

STOWER, M.J. & HARDCASTLE, J.D. (1985). The results of 1,115 patients with colorectal cancer treated over an 8 year period in a single hospital. *Eur. J. Surg. Oncol.*, **11**, 119.

WOLLEY, R.C., SCHREIBER, K., KOSS, L.G., KARAS, M. & SHERMAN, A. (1982). DNA Distribution in human colon carcinomas and its relationship to clinical behavior. *J. Natl Cancer Inst.*, **69**, 15.